# Access the Unified Health System actions and services from the perspective of judicialization^1^

**DOI:** 10.1590/1518-8345.1012.2689

**Published:** 2016-05-03

**Authors:** Raquel de Souza Ramos, Antonio Marcos Tosoli Gomes, Denize Cristina de Oliveira, Sergio Corrêa Marques, Thelma Spindola, Virginia Paiva Figueiredo Nogueira

**Affiliations:** 2 PhD, RN, Instituto Nacional de Câncer José Alencar Gomes da Silva, Rio de Janeiro, RJ, Brazil; 3 PhD, Full Professor, Faculdade de Enfermagem, Universidade do Estado do Rio de Janeiro, Rio de Janeiro, RJ, Brazil; 4 PhD, Adjunct Professor, Faculdade de Enfermagem, Universidade do Estado do Rio de Janeiro, Rio de Janeiro, RJ, Brazil; 5 PhD, Associate Professor, Faculdade de Enfermagem, Universidade do Estado do Rio de Janeiro, Rio de Janeiro, RJ, Brazil; 6 Doctoral student, Faculdade de Enfermagem, Universidade do Estado do Rio de Janeiro, Rio de Janeiro, RJ, Brazil. Scholarship holder from Coordenação de Aperfeiçoamento de Pessoal de Nível Superior (CAPES), Brazil

**Keywords:** Unified Health System, Health Policy, Right to Health, Judicial Decisions, Social Perception, Nursing

## Abstract

**Objective:**

the judicialization of health is incorporated into the daily work of health institutions in Brazil through the court orders for access. In this study, the objective was to describe the contents of the social representations of access, through judicialization, for the health professionals.

**Method:**

qualitative study based on Social Representations Theory, involving 40 professionals, at a teaching hospital and at the center for the regulation of beds and procedures in Rio de Janeiro. Forty semistructured interviews were held, to which the thematic-categorical content analysis technique was applied.

**Results:**

the health professionals' attitude towards the reality the judicialization imposes is negative, but they acknowledge this resource as necessary in view of the public health crisis. Judicialization is considered a strategy to exercise citizenship that superimposes individual on collective law, increases social inequalities in access and compromises the efficacy of health policies.

**Conclusion:**

considering social representation as a determinant of practices, the representations that emerged can contribute to the change of the professionals' practices. Improvements in user care should be promoted, characterized as one of the main challenges to advance in universal access to health.

## Introduction

The judicialization of health is increasingly present in the daily work of public health institutions in Brazil. This expression has been incorporated into the public debate, leading to multiple uses and meanings. The theme has been hardly explored in Nursing studies, being a theoretical-empirical gap that needs urgent completion. Judicialization is considered a recent phenomenon, constituted by the influence of the Judicial Power in political and social institutions. In Brazil, the theme started to be studies after the enactment of the 1988 Federal Constitution^(^ 1 ^)^, which affirms in article 196 that health is a right of all and a duty of the State^(^ 2 ^)^.

The combination between the democratic failure that marks many countries in Latin America and favorable opportunities in courts (like the existence of constitutional resources of protection, lax requisites for their legal processing and the solution speed) has lead to the increased judicialization of the right to health in different countries^(^ 2 ^-^ 3 ^)^. If, on the one hand, there is some evidence that the court cases private persons started to claim individual rights can benefit the middle class further than poorer groups, the legal execution of the principles and standards of health rights can also favor the strengthening of the health system and universal coverage^(^ 3 ^)^, as the transformation, upon court order, of the legal system and the unification of benefits demonstrate, like in Colombia for example^(^ 4 ^)^.

Another observation that stands out is that most countries that adopt the logic of a universal health system do not guarantee the right to health, but the right to health services. This nuance grants the Brazilian health system quite peculiar characteristics, as the protected good is the right to health. Thus, the judicialization of health in Brazil differs from the international picture of this particular aspect^(^ 1 ^,^ 4 ^)^.

The universal access to health in Brazil, although guaranteed in the constitution, is not always complied with in all situations that claim it and, thus, an increasing number of citizens turn to the judiciary power to claim the right to health and to guarantee the access to the demands the system does not receive. In that context, the fact of turning to legal instances to get access to what is guaranteed in the constitution represents a great paradox, which considering that the health policy is developed in a State of Right, with a broad democratic space, involving the Civil Society and the Legislative, Executive and Judiciary Powers in the decisions and discussions on where the Brazilian Health Policy is heading.

In the professional practices, a weak link is perceived between users and health services. The weak support of the services and the neglect in daily care can also culminate in the search for legal entities to put in practice the right to health, that is, in the operation of the system, the lack of complex and high-cost services can sometimes produce conflicting relations among professionals, users and health services.

The judiciary power has commonly received claims from Unified Health System (SUS) users to get access to the resources needed to recover their health, such as medicines that are costly or do not figure on the official list of standardized dispensation by the official public entities; hospitalization in high-complexity and costly beds (intensive care and specialty beds, such as cancer care for example) and to claim medical care, through outpatient appointments and diagnostic and therapeutic procedures^(^ 1 ^,^ 3 ^)^.

The use of the courts as the main means to guarantee access, where the judiciary system starts to serve as yet another entry door to the system, can mischaracterize the widely discussed theoretical and conceptual bases on the access to the Brazilian public health system.

In view of the above reflections, the objective in this study was to analyze the judicialization process of health in the context of the SUS for managers, regulators and health professionals.

## Method

Qualitative study based on Social Representations Theory^(^ 5 ^-^ 6 ^)^, developed in two research locations: a public state-owned teaching hospital that delivers medium and high-complexity care and that executes the court orders for diagnostic and therapeutic procedures and hospitalizations; and the state-owned center for the regulation of health services, both located in the city of Rio de Janeiro.

These services were chosen in function of an earlier study on the practical feasibility, where it was identified that court orders are frequently attended to, thus permitting the identification of the group's social representation on the object.

The subjects were chosen based on the following inclusion criteria: being a member of the institution's fixed staff, having worked in the context for more than one year and having experienced situations that involve the judicialization of health in professional practice.

The data were collected in 2013 through a sociodemographic questionnaire to characterize the subjects and in-depth semistructured interviews, guided by a thematic script that addressed the judicialization of health, the subjects' experiences, the work process to respond to the court orders, the interviewees' position towards the research problem, the health team's relationship with the patients and with the family who entered the system by court order, the impact of the legal measures on the planning of the health policy, the main demands and operational difficulties to get care upon court order, the correlation between the judicialization of health and the SUS principles; the participants' perception on the access to the SIS without the court orders and the feelings, images and attitudes in the context of the judicialization in health.

Forty interviews were held, including 17 professionals who were technically working, 17 managers and six regulators. The testimonies were recorded, transcribed and then submitted to the thematic content analysis^(^ 7 ^)^, operated in the software QSR Nvivo, version 10.0.

The study was submitted to the Research Ethics Committee at the University Hospital Pedro Ernesto at *Universidade do Estado do Rio de Janeiro* and approved under Opinion CAAE 14933613.1.0000.5259, issued on 04/26/2013.

## Results

### The study group

The descriptive analysis of the study participants showed that 34 interviewees worked in hospitals, equally divided between professionals active in direct care delivery and local managers. Six participants worked at the regulation center. The latter group is smaller due to changes in the structure of the regulation center in the State of Rio de Janeiro which, at the time of the data collection, was changing the form of hiring, so that only six regulatory physicians existed. As a result of this distribution, subjects were approached who were practical observers (17 managers), as well as subjects who delivered professional care activities directly (17 individuals) and indirectly (6 regulatory physicians) s) to the users who entered the system by court order, which permits a broader look on the research problem.

The largest professional category were nurses (19 professionals), followed by physicians (12 testimonies), four nursing technicians and five social workers. Thus, 10% were secondary-level professionals, while 90% held a higher education degree. Also regarding this variable, the data appoint that 57.5% were nursing professionals (involving nurses and nursing technicians), while 42.5% came from other professional categories.

As regards the gender distribution, 34% were male and 66% female. The largest age range corresponded to subjects between 36 and 40 years of age, with 20% of the subjects. In addition, testimonies between 46 and 50 years and between 31 and 35 years of age stood out. Only 9% were professionals over 56 years of age, which can be due to the retirement benefits granted as from the age of 55 years, according to the length of the contribution to social security.

In this respect, 34% of the subjects were starting their career, having worked between one and five years. On the other hand, 37% had been professionally active for more than 16 years, indicating a group with consolidated professional experience.

### Social representations of judicialization

The data analysis resulted in 16 themes and 481 registration units. Initially, the testimonies highlighted that the use of the legal route to claim medicines, hospitalization beds, care materials, among other health demands, is associated with the higher information level of the population who goes to court.

Judicial measures allow the subjects to present their viewpoints publicly, turning judicialization into a more accessible discussion channel for everyone, mainly when the debate takes place in a field in which citizens do not have a majority decision-making position^(^ 8 ^)^. It is highlighted that most favorable decisions in health judicialization benefit users who are socially better off, contributing to the worsening of social injustices in terms of access to health products and services, as people in better socioeconomic conditions have greater access to the Judiciary Power, thus characterizing a mechanism that promotes distributive injustice^(^ 9 ^)^.

In the democratic context of the current political scenario, the judicialization of health refers to the claims and legitimate action modes of citizens and institutions, employed to guarantee the promotion of the rights to citizenship, guaranteed by the Brazilian and international legal apparatus^(^ 10 ^)^.

*This type of access is still elite but, little by little, everyone is exercising his citizenship further, without depending on the favors, paternalism and assistance of the State* (Interview 13-Physician).

*It is an exercise of citizenship, it tends to elaborate the common citizen's critical assessment further* (Interview 24-Nurse).

The legal measures tend to express one of the strategies to manifest citizenship. The users' demands are heard and their needs and expectations are attended to, which are not always welcomed when in contact with the health services. The court decisions turn into an expression of the citizens' voice and power over their health, which are not always accepted in environments where the health professionals' hegemony prevails in the conduction of clinical cases. These disagreements and mismatches, sometimes mediated by precarious communication, are fertile ground for the dissemination of legal actions in health.

Through judicialization, it is observed that the legal measures tend to individualize the policy, privileging individual over collective rights.

*First, only one person is attended to, it is difficult for a court order to benefit the group. Second, you surpass others who are queuing for the some resource, that's individual rights prevailing over collective rights* (Interview 5-Physician).

The above testimony ratifies experts' position, who affirm that judicialization is a strategy the citizens adopt to safeguard their rights, initially employed individually, and more recently in the collective dimension^(^ 11 ^)^. The prevalence of individual over collective rights stands out when observing that collective were replaced by individual historical forms of struggle, through the use of the legal route, in which the Judiciary Power is acknowledged as an ally of citizenship^(^ 12 ^)^ **_._** Nevertheless, the prevalence of merely legal and not political language can harm the group, management and the implementation of policies at the different health levels and contexts^(^ 1 ^,^ 12 ^)^.

Thus, the increasing judicialization of health actions increases the inequalities among citizens in the use of health services, and also compromise the efficacy of public policies through the need to reallocate funds to comply with the judicial claims^(^ 8 ^,^ 11 ^)^.

The judicial demands, mostly individual, are related to health procedures and inputs, such as medicines, exams and surgeries, whose technologies have not been incorporated yet into the list of procedures funded by the SUS.

*Pharmaceutical care even receives decisions to provide medicines beyond the scope of the Ministry and I've seen a case of a Brazilian woman who lived in Canada and left her son and husband there and came back to Brazil to get treated in Rio de Janeiro, using a drug beyond the scope there and here, because she knew she had very big chances of getting the drug here in court* (Interview 5-Physician).

Today, pharmaceutical care represents one of the main judicialized resources, as the speed of discoveries, with consequent advances in health technology, is not the same observed in the incorporation of these resources by the SUS^(^ 10 ^)^. On the other hand, the incorporation of new technologies without any critical sense, which the judicialization encourages, can originate in the strong influence large pharmaceutical industries exert on the State and the health professionals, mainly physicians.

*On the other hand, other claims find no scientific support but rest on commercial interests of the pharmaceutical industry* (Interview 30-Nursing Technician).

The high costs of the new drugs influence pharmaceutical care in the SUS, as the court cases oblige public entities to supply the drugs, based on the constitutional premise of universal access to health. Through these decisions, the judiciary power contributes to the introduction of unstandardized medicines in the SUS, as well as drugs that are not part of the Ministry of Health's established protocols^(^ 13 ^)^.

The influence of the pharmaceutical industry in this scenario reveals that drug commercials can influence medical prescriptions, demands and needs deriving from the medicalization of society. These drugs tend to be costlier and not always offer advantages over others already available on the market^(^ 14 ^-^ 15 ^)^. Therefore, these court decisions cause disorganization in public management, as they limit the ability to plan, compromising the administrative efficiency in user services^(^ 8 ^,^ 14 ^)^.

Claims for inpatient beds are increasingly frequent, mainly for intensive care and surgical procedures. There are important bottlenecks in the medium and high-complexity and high-cost care lines, which make it difficult to deliver integral care in the SUS. Although the system offers the resources universally, it is unable to attend to all demands^(^ 16 ^)^.

*Something else is the intensive care bed, as the number of beds will never be enough to attend to the demand of the entire population, which is growing a lot, there is a repressed demand in the number of intensive care beds, so, often, the relative needs to use the judiciary power to be able to hospitalize his family member* (Interview 2-Nurse).

Hospitalization in intensive care beds happens based on the determination of the risk level a specific disease offers for the person's life. This indication is technical and respects protocols with a view to indications that are safe for the users, besides optimizing the use of this scarce resource in the public network. Technical criteria are not always employed when these beds are claimed in court, as these requests are associate with some professionals' lack of experience and even with the family's desire which, for cultural reasons, believes that hospitalization in these services is the best care alternative.

*In one situation, the patient was hospitalized upon court order, he was discharged but his grandson did not accept it, he wanted him to be kept here, because he saw that the structure is good, that there's always someone at his side* (Interview 6-Nurse).

The above issued evidence that the social representation of quality in health rests on technology, mobilizing the population to seek judicial access to health^(^ 17 ^)^. The media associates the image of technology with quality in health. This can be observed in the commercials disseminated in the media, when they present new "health products", such as supplementary health insurances, presentations of new hospitals, despite knowing that hard technology does not always guarantee the quality of care^(^ 17 ^)^.

In that sense, the social representation about the judicialization of health seems to be related with the representations of the SUS and the hospital, strengthening the hospital-centered ideology. Hence, this representation of the SUS seems to influence the judicial demand for high-complexity and technologically novel hospitals, beds, procedures, inputs and medicines, which are not always available in the public health network.

Despite acknowledging the harm of individualized policies, resulting from the phenomenon of judicialization, the professionals affirm that, at some moment along their professional trajectories, they ended up stimulating the search for legal help in cases in which they perceived that the difficulties the system faced could impair the user's therapeutic proposal, appointing tension between positivity and negativity towards the study object.

*But for who is about to watch a person die, we really move to the judiciary power and that strengthens a process we are against, we end up strengthening it* (Interview 24-Nurse).

This position can reveals the health professionals' social and ethical commitment to the users, as they acknowledge the need to turn to this instance as a way to guarantee the right to health. The knowledge about most public health services' conditions evidences the need to reformulate the management and health care practices, in which the professionals can replace the ingrained institutional cultures that tend to "thingify" the health service users in order to attend to their needs^(^ 18 ^)^. The professionals' encouragement of judicialization can constitute one of the ways to combat institutional cultures that perpetuate the disqualification of the health policy^(^ 18 ^)^.

The professionals' attitude is justified by the slow administrative routes that are sometimes insensitive to the users' needs, as opposed to the judiciary power, which is fast in this service^(^ 19 ^)^. Hence, the action of the Judiciary Power in health happens to solve the State's difficulties to attend to the population's needs. Therefore, these actions derive from different difficulties the users experience in the public health system, appointing that the judicialization of health is not a problem by itself, but results from a range of difficulties and problems constructed over the past decades^(^ 1 ^,^ 3 ^,^ 10 ^)^.

One of the main challenges the SUS faces is its own practice. In that sense, the users, professionals and managers have attempted to keep the legal right to health coherent with what happens in practice^(^ 20 ^)^. In this search for coherence, the population has claimed for changes, whether through legal updates and claims for current legislation to be put in practice.

Another point that should be highlighted is that the main target, as well as the main challenge of the SUS - universal coverage - guarantees health for all. As a big construction, deriving from one of the main social movements in the country - the Health Reform - this model is taking form and is facing different difficulties that drive the wheels of judicialization in Brazil.

Nevertheless, the actions of the Judiciary Power are extremely important to put in practice the right to citizenship and to fully access and use the right to health. On the opposite, this intervention is characterized as a point of tension between policy makers and executors, who find themselves obliged to guarantee social rights, with some cases disagreeing from the policy and surpassing the budgetary possibilities.

In the context of citizenship, the judicialization of health, according to the participants, may not attend to the interests of the group, as the decisions tend to focus on punctual situations that do not represent the collective citizenship rights, besides burdening the public administration and without resulting in better conditions of the health system or in the concretization of citizenship^(^ 21 ^-^ 22 ^)^. Hence, on the one hand, the judicialization of health announces the possibility of putting in practice the right to health but, on the other, turns into a risk for the universal nature of the public policy, unless well-defined criteria are adopted for the technical appropriateness and budgetary feasibility of the claim^(^ 1 ^)^.

## Conclusion

The results appoint a socio-cognitive reconstruction of the predominantly negative judicialization process that is consolidating, in which the limitations of the actual SUS are observed that initiate the judicialization movement. The concrete dimension expresses that the activities of the judiciary, despite positive end results, guaranteeing universal access to a certain resource for the claimants, receive an overall negative assessment from the research participants.

The judicialization process influences the health professionals' practice because these professionals witness the deepening of inequalities, the worsening of people who do not get access to a certain therapeutic resource in function of the obligation to immediately comply with a court order, which is not always technically indicated. This tends to grant concrete shapes to feelings of injustice experienced in the social group studied and which are expressed in their discourse.

For the group studied, in a macropolitical context, the judicialization process seems to jeopardize the premises of the SUS, to the extent that most actions do not benefit the group nor respect the guidelines of the system, tending to promote the mischaracterization of the SUS and consequently impairing universal health coverage in the country.

In view of the above, it is concluded that the participants understand the judicialization as a strategy to exercise citizenship that superimposes the individual on the collective, increases social inequalities in universal access and compromises the efficacy of the health policies.

It is important to highlight that this study comes with limitations, as the users who go to court and the judiciary professionals were not investigated. Therefore, it would be interesting for new studies to be developed that address these two social groups.

In addition, efforts need to be made to promote the strengthening and enrichment of the debate on the strategies to guarantee the practice of the right to health and the universal access to health actions, involving health and judiciary professionals, users and researchers on theme so that, based on evidence, they can devise proposals to solve the claims, mainly the most recurring ones, moving together towards the advance of democracy and citizenship and the improvement of access and universal coverage in the SUS.
